# *Helicobacter pylori* reduces METTL14-mediated VAMP3 m^6^A modification and promotes the development of gastric cancer by regulating LC3C-mediated c-Met recycling

**DOI:** 10.1038/s41420-025-02289-z

**Published:** 2025-01-18

**Authors:** Xixi Cui, Mingjie Chang, Yuqiong Wang, Jiayi Liu, Zenghui Sun, Qiyu Sun, Yundong Sun, Juchao Ren, Wenjuan Li

**Affiliations:** 1 Key Laboratory for Experimental Teratology of Chinese Ministry of Education, The Shandong Provincial Key Laboratory of Infection and Immunology, Department of Pathogenic biology, School of basic medical sciences, Cheeloo College of Medicine, Shandong University, Jinan, PR China; 2 Department of Urology, Qilu Hospital, Shandong University, Jinan, PR China

**Keywords:** Gastric cancer, Diagnostic markers

## Abstract

*Helicobacter pylori* (*H. pylori*) plays an important role in the malignant transformation of the gastric mucosa from chronic inflammation to cancer. However, the mechanisms underlying the epigenetic regulation of gastric carcinogenesis mediated by *H. pylori* remain unclear. Here, we uncover that *H. pylori* inhibits METTL14 by upregulating ATF3. METTL14 inhibits gastric cancer (GC) cell proliferation and metastasis in vitro and in vivo. Downregulation of METTL14 inhibits Vesicle-associated membrane protein-3 (VAMP3) by reducing the m^6^A modification level of VAMP3 mRNA and the stability of IGF2BP2-dependent mRNA. *H. pylori* also accelerates the malignant progression of GC by regulating VAMP3/LC3C-mediated c-Met recycling. Moreover, the expression of METTL14 and VAMP3 in *Hp+* chronic gastritis tissues is much lower than that in *Hp−* chronic gastritis tissues. METTL14 and VAMP3 expression levels are downregulated notably in cancerous tissues of patients with GC. Therefore, our results show a novel METTL14-VAMP3-LC3C-c-Met signalling axis in the GC development mediated by *H. pylori* infection, which reveals a novel m^6^A epigenetic modification mechanism for GC and provides potential prognostic biomarkers for GC progression.

## Introduction

Gastric cancer (GC) is one of the most common cancers worldwide. Its incidence and mortality ranked the fifth in the world in 2022, with the highest incidence in East Asia and Eastern Europe [1]. The high morbidity and mortality of GC significantly threaten human life. *H. pylori* colonizes the epithelial cells of the gastric mucosa, and long-term infection leads to chronic nonatrophic gastritis, a persistent inflammation that leads to gastric mucosal atrophy and intestinal metaplasia, followed by dysplasia and eventually malignant transformation [2]. *H. pylori* produces many different virulence factors, of which cytotoxin-associated gene A (CagA) is considered to be an important pathogenic factor. CagA activates multiple intracellular pathways, such as the Shh, Wnt, c-Met and Ras/Raf signalling pathways, that control cell proliferation and migration, ultimately leading to malignant transformation [3]. However, the malignant transformation of cells caused by *H. pylori* involves a regulatory network composed of host epigenome, transcriptome and proteome, the specific mechanism of which has not been elucidated [4].

N6-methyladenosine (m^6^A) is the most common posttranscriptional modification in eukaryotes. m^6^A modification is a dynamic and reversible process that is jointly regulated by m^6^A methyltransferases (METTL3, METTL14 and WTAP), m^6^A demethylases (FTO and ALKBH5) and m^6^A readers (YTHDF1/2/3, YTHDC1, IGF2BP1/2/3 and so on) [5]. In the m^6^A methyltransferase complex, METTL3 functions as a catalytic subunit, and METTL14 functions as an RNA-binding scaffold that recognizes the substrate and promotes METTL3 stabilization [6]. IGF2BPs play an important role in protecting mRNA from degradation and promoting its stability [7]. There is growing evidence that the dysregulation of m^6^A-related proteins and downstream target m^6^A modification mRNA significantly affect the malignant transformation of human cancers, including colorectal cancer [8], pancreatic cancer [9], hepatocellular carcinoma [10] and gastric cancer [11]. It is reported that GC cells with *H. pylori* infection have higher m^6^A modification level [12]. However, the mechanisms by which the aberrant expression of these m^6^A-related molecules in the process of *H. pylori*-mediated malignant transformation of gastric mucosa still need to be elucidated.

Vesicle-associated membrane protein-3 (VAMP3) is a ubiquitously expressed vesicular SNARE protein that recycles specific receptors to and from the plasma membrane through the recycling endosome (RE) [13]. VAMP3 mediates the recycling of the transferrin receptor, transferrin and integrins to the plasma membrane. It is also involved in granule transport in platelets [13, 14]. In neuroblastoma cells, miR-124 promotes cell proliferation and inhibits cell apoptosis by suppressing the expression of VAMP3 [15]. Therefore, VAMP3 is of crucial importance in efficient intracellular transport and may play an important role in the occurrence and development of cancer.

Our study demonstrates that *H. pylori* reduces METTL14-mediated VAMP3 m^6^A modification and promotes the development of GC by regulating LC3C-mediated c-Met recycling.

## Results

### *Helicobacter pylori* infection downregulates METTL14 in GC cells

**Fig. 1:**
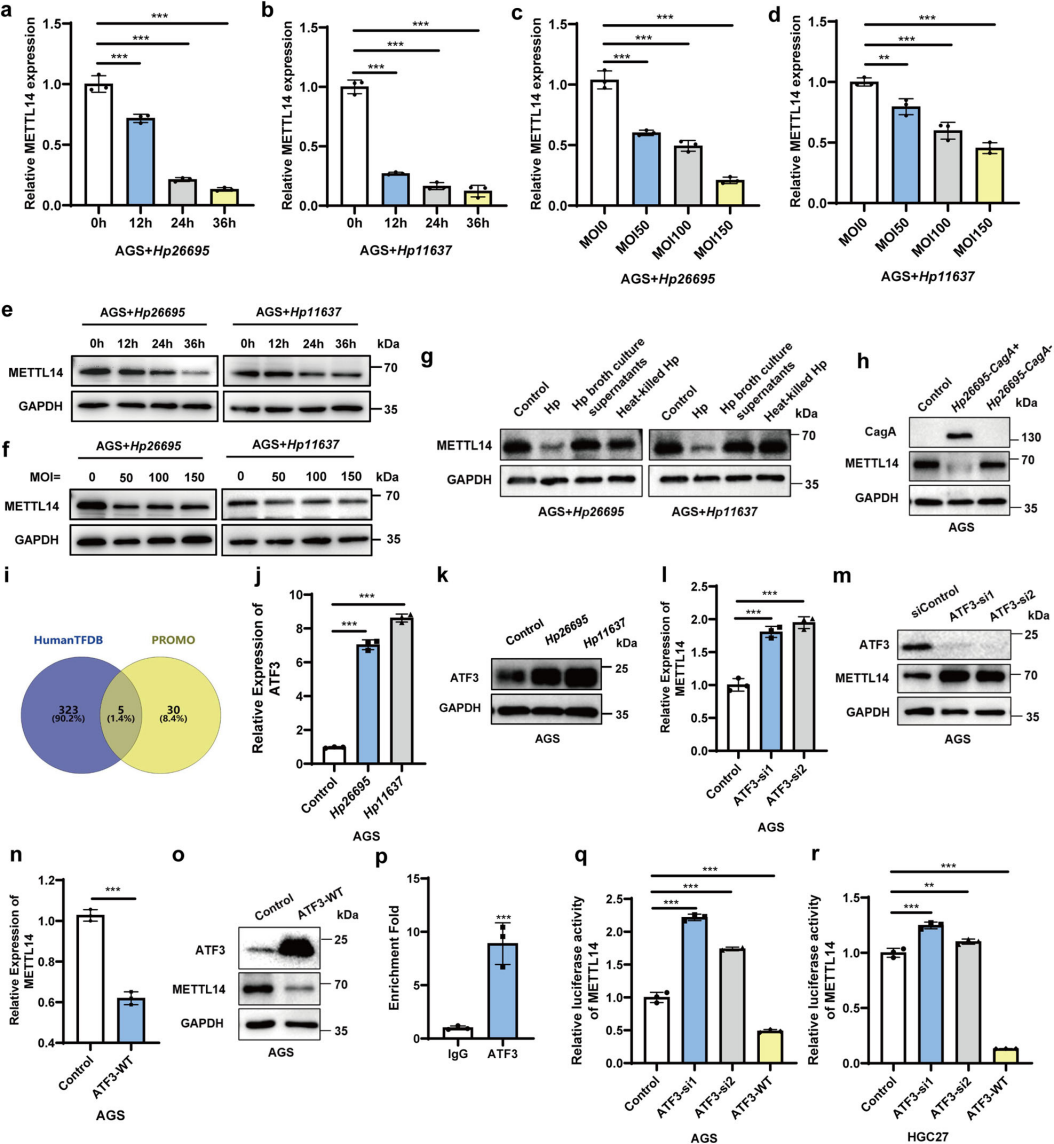
*Helicobacter pylori* infection downregulates METTL14 in GC cells. **a**–**d** METTL14 mRNA was measured by qRT‒PCR in AGS cells treated with *Hp26695* and *Hp11637* at different time points and different MOIs. **e**, **f** METTL14 protein in AGS cells treated with *Hp26695* and *Hp11637* at different time points and MOIs was detected by Western blot. **g** Western blotting was used to detect METTL14 expression levels in AGS cells treated with *H. pylori*, *H. pylori* broth culture supernatants and heat-killed *H. pylori* at MOI = 100 for 12 h. **h** METTL14 protein levels in AGS cells treated with *Hp26695-CagA+* strain and *Hp26695-CagA-* strain at MOI = 100 for 12 h were detected by Western blot. **i** Venn diagram showing that HumanTFDB and JASPAR have five overlapping transcription factors for METTL14. **j**, **k** qRT‒PCR and Western blot analyses of ATF3 in AGS cells treated with *Hp26695* and *Hp11637* at MOI = 100 for 12 h. **l**, **m** METTL14 mRNA and protein levels were measured by qRT‒PCR and Western blot in ATF3-knockdown AGS cells. **n**, **o** METTL14 mRNA and protein were measured by qRT‒PCR and Western blot in ATF3-overexpression AGS cells. **p** ChIP assays were used to determine the binding of ATF3 to the METTL14 promoter in HGC27 cells. **q**, **r** A dual-luciferase reporter assay was used to determine the activity of the METTL14 promoter (1000 bp) in AGS and HGC27 cells with ATF3 overexpression or knockdown. *p < 0.05, **p < 0.01, ***p < 0.001.

To investigate the role of m^6^A modification in the development of *H. pylori*-mediated GC, we detected the effect of *H. pylori* on the expression of m^6^A modulators in our previous study and found that *H. pylori* significantly downregulated the expression of the m^6^A core methyltransferase METTL14 [16]. To explore the molecular mechanism by which *H. pylori* downregulates METTL14, we infected AGS cells with *Hp26695* and *Hp11637* at specific times and concentrations and detected the expression of METTL14. *H. pylori* could significantly downregulate METTL14 when the bacteria had infected AGS cells for 12 h, and the downregulation trend was more significant with a longer infection time (Fig. 1a, b, e). Infection of AGS cells with *H. pylori* at concentrations of MOI = 50, 100 and 150 also significantly downregulated METTL14 (Fig. 1c, d, f). Meanwhile, to identify which component of *H. pylori* downregulates METTL14 expression, we infected GC cells with *H. pylori*, *H. pylori* broth culture supernatants and heat-killed *H. pylori*. *H. pylori* broth culture supernatants and heat-killed *H. pylori* could not downregulate METTL14 (Fig. 1g), indicating that the downregulation of METTL14 induced by *H. pylori* might depend on the interaction between living bacteria and cells. *H. pylori* produces many different virulence factors. CagA, an important oncogenic factor, activates the Ras, Raf and ERK signalling pathways, which control cell proliferation [3]. Therefore, we speculated that CagA could downregulate METTL14. We found that METTL14 was significantly downregulated by *Hp26695-CagA+* strain but not by *Hp26695-CagA-* strain (Fig. 1h).

To further explore the molecular mechanism by which *H. pylori* downregulates METTL14, we first predicted the potential transcription factors that may bind to the promoter regions of METTL14 through the online bioinformatics tools HumanTFDB and JASPAR (Fig. 1i). We found that ATF3 was most significantly upregulated in AGS cells treated with *Hp26695* and *Hp11637* (Fig. 1j, k and Supplementary Fig. 1a). METTL14 expression was significantly increased when ATF3 was knocked down (Fig. 1l, m). Conversely, ATF3 overexpression significantly reduced METTL14 expression (Fig. 1n, o). To verify whether ATF3 is a transcription factor for METTL14, chromatin immunoprecipitation (ChIP) assays were employed, and the results revealed that ATF3 bound to the METTL14 promoter (Fig. 1p). Furthermore, a dual-luciferase reporter assay confirmed that ATF3 knockdown significantly enhanced METTL14 luciferase activity and that ATF3 overexpression significantly decreased METTL14 luciferase activity in AGS and HGC27 cells (Fig. 1q, r). These results indicate that ATF3 is a transcriptional repressor for METTL14.

### METTL14 inhibits GC cell proliferation and metastasis in vitro and in vivo

**Fig. 2:**
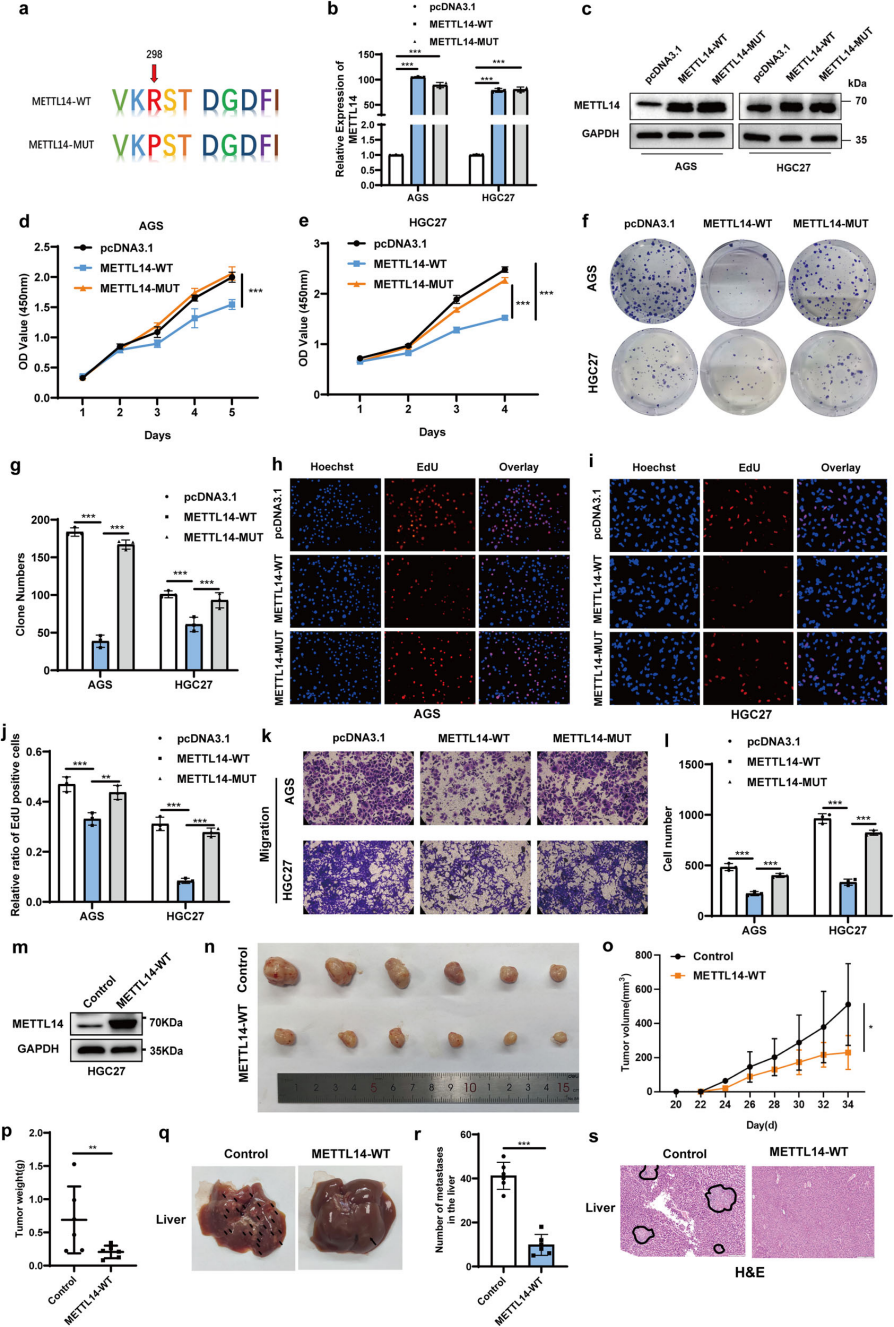
METTL14 inhibits GC proliferation and metastasis in vitro and in vivo. **a** Schematic representation of the METTL14-R298P mutant plasmid. **b, c** qRT‒PCR and Western blotting were used to detect METTL14 expression levels in AGS and HGC27 cells with wild-type or catalytic mutant METTL14 overexpression. CCK-8 (**d, e**), colony formation (**f**) and EdU (**h, i**) assays were used to detect the effect of wild-type or catalytic mutant METTL14 overexpression on GC cell proliferation. Quantification of the colony formation (**g**) and EdU (**j**) assays. Transwell assays (**k**) were used to detect the effect of wild-type or catalytic mutant METTL14 overexpression on GC cell migration. Quantification of the Transwell assay (**l**). **m** METTL14 protein was detected by Western blot in HGC27 cells infected with wild-type METTL14 lentivirus. **n** Image of tumours from xenograft mouse models in METTL14-WT group (*n* = 6) and control group (*n* = 6). **o, p** The tumour volumes and weights were measured. **q, r** Images of the metastatic nodes in the livers and quantification of the metastatic nodes (**r**). **s** H&E-stained liver sections. *p < 0.05, **p < 0.01, ***p < 0.001.

To examine the function of METTL14 in GC cells, we directly knocked down METTL14 using two shRNA plasmids (Supplementary Fig. 2a, b) and found that METTL14 knockdown significantly promoted the proliferation, clonogenic and migration abilities of GC cells (Supplementary Fig. 2c-k). The Arg298 site of METTL14 can interact with METTL3. Mutation of this site significantly reduces methyltransferase activity, and the m^6^A methyltransferase complex can no longer distinguish the cognate RNA target from the mutant RNA substrate [17]. Therefore, wild-type METTL14 and METTL14-R298P mutant plasmids were constructed (Fig. 2a–c). The proliferation and migration of GC cells were decreased with the overexpression of wild-type METTL14. Notably, compared to wild-type METTL14, expression of the METTL14-R298P mutant lost the ability to inhibit the proliferation and migration of GC cells (Fig. 2d–l).

To detect the effects of METTL14 on the growth and metastasis of GC cells in vivo, we overexpressed METTL14 in HGC27 cells with wild-type METTL14 lentivirus (Fig. 2m). A subcutaneous injection test indicated that the METTL14 overexpression significantly inhibited tumour growth (Fig. 2n). The weight and volume of the tumours in the wild-type METTL14 group were significantly lower than those in the control group (Fig. 2o, p). In a tail vein injection assay, we observed that the METTL14-WT group had fewer metastatic nodules in the liver than the control group (Fig. 2q–s). Taken together, these results demonstrate that METTL14 efficiently inhibited GC cell proliferation and metastasis in vitro and in vivo.

### *H. pylori* induces differential m^6^A modification in GC cells

**Fig. 3:**
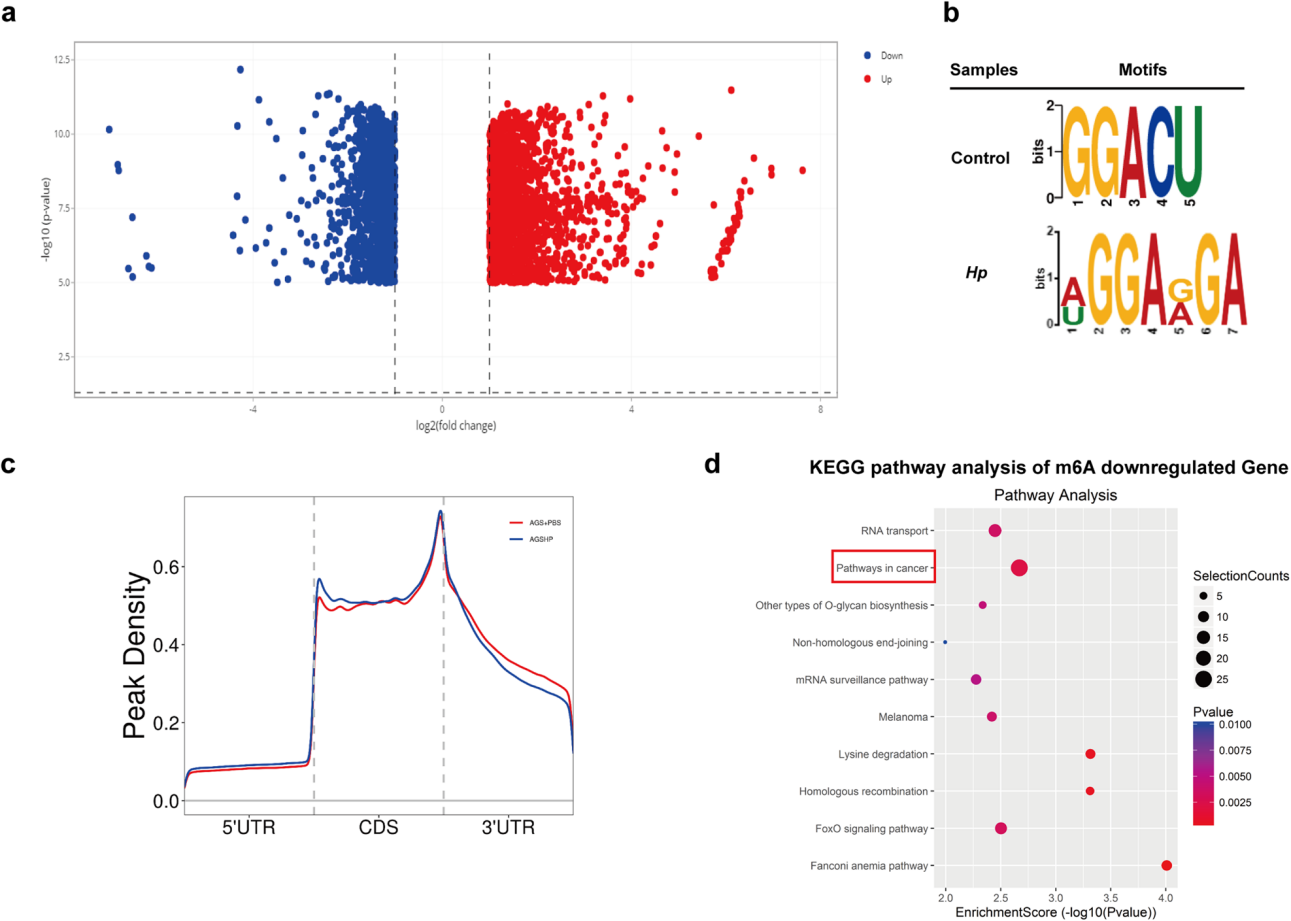
*H. pylori* induces differential m^6^A modification in GC cells. **a** Volcano plots were constructed to determine the m^6^A modification levels of differentially expressed m^6^A-modified genes at *p < 0.05 in AGS cells treated with *Hp26695* at MOI = 100 for 12 h; blue indicates genes with downregulated m^6^A modification, and red indicates genes with upregulated m^6^A modification. **b** m^6^A consensus motifs with the lowest p values detected by DREME. **c** Proportions of m^6^A peak distribution in the 5′UTR, CDS and 3′UTR regions of mRNA transcripts. **d** KEGG pathway analysis of m^6^A peak-downregulated genes in AGS cells treated with *Hp26695* compared with the control. *p < 0.05, **p < 0.01, ***p < 0.001.

To investigate the variations in m^6^A modifications in specific genes, we performed m^6^A-modified RNA immunoprecipitation sequencing (MeRIP-seq) in *H. pylori*-treated AGS cells. MeRIP-seq identified a total of 3457 genes with significant differences in m^6^A levels in *H. pylori*-treated AGS cells. Among these genes, the m^6^A levels of 1981 genes were significantly upregulated, and the m^6^A levels of 1476 genes were significantly downregulated (Fig. 3a). The m^6^A consensus motif of GGACU was highly enriched in the control group, while a WGGARGA (W = A/U, R = G/A) motif was highly enriched within m^6^A sites in *H. pylori*-treated cells (Fig. 3b). Moreover, MeRIP-seq suggested that m^6^A peaks were especially abundant in the CDS and 3′UTR regions of mRNA transcripts in both control and *H. pylori*-treated cells (Fig. 3c).

KEGG pathway enrichment analysis was performed on the differential m^6^A genes to understand their major participating signalling pathways, and it was found that the m^6^A downregulated genes were mainly involved in cancer pathways (Fig. 3d). These results suggest that *H. pylori* infection can induce differential m^6^A modification in GC cells.

### METTL14-mediated m^6^A modification of VAMP3 mRNA maintains its IGF2BP2-dependent stability

**Fig. 4:**
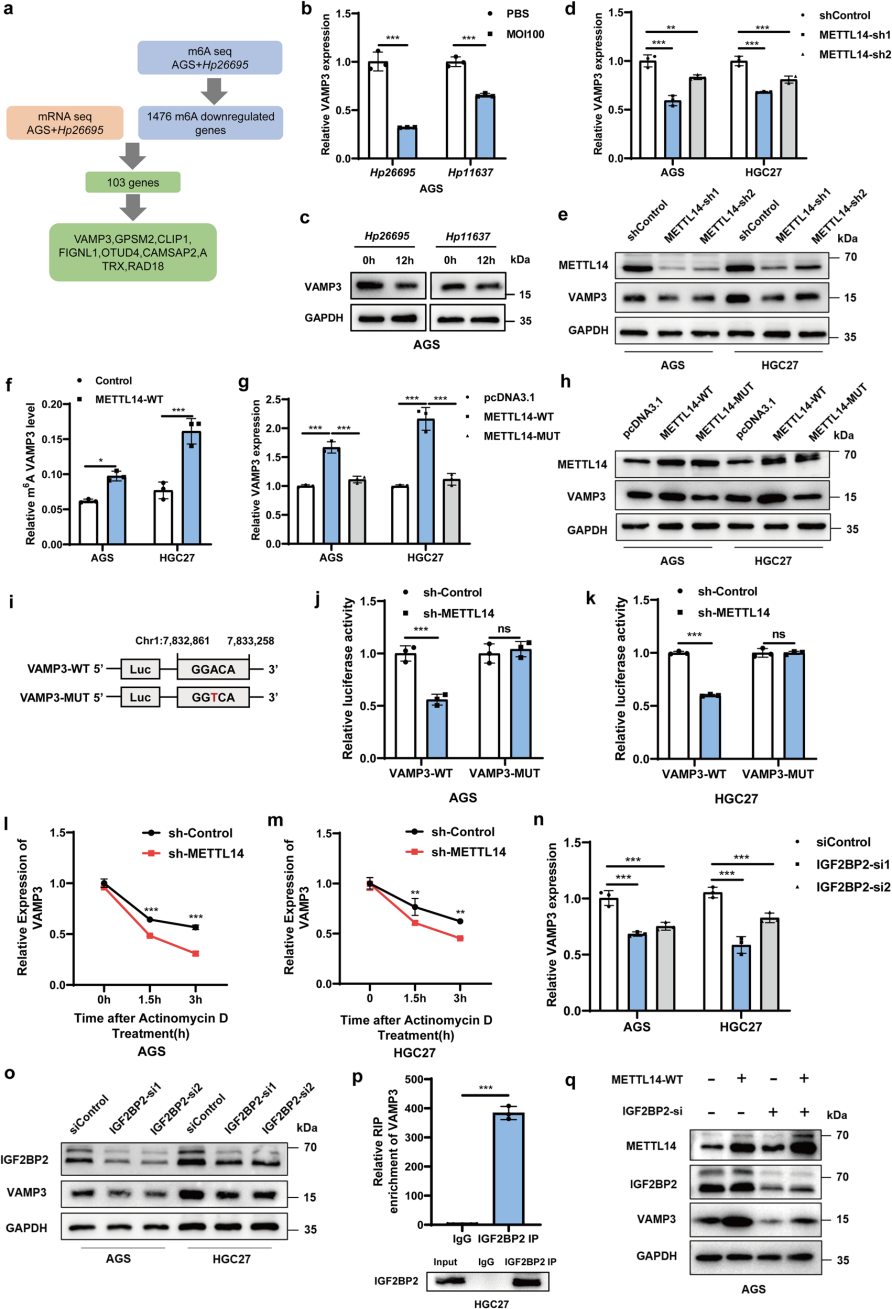
The METTL14-mediated m^6^A modification of VAMP3 mRNA maintains its IGF2BP2-dependent stability. **a** Flow chart of METTL14 downstream target gene screening. **b**, **c** VAMP3 mRNA and protein expression levels were measured by qRT‒PCR and Western blot in AGS cells treated with *Hp26695* and *Hp11637* at MOI = 100 for 12 h. **d**, **e** VAMP3 mRNA and protein expression levels were measured by qRT‒PCR and Western blot in AGS and HGC27 cells with METTL14 knockdown. **f** MeRIP-qPCR was used to detect the m^6^A modification level of VAMP3 in AGS and HGC27 cells with METTL14 overexpression. **g**, **h** VAMP3 mRNA and protein expression levels in AGS and HGC27 cells treated with wild-type or catalytic mutant METTL14 overexpression were measured by qRT‒PCR and Western blot. **i** Schematic representation of the VAMP3 m^6^A site wild-type dual-luciferase plasmid and the m^6^A site mutant dual-luciferase plasmid. **j**, **k** A dual-luciferase reporter assay was used to determine the activity of the wild-type or mutant VAMP3 firefly luciferase reporter in AGS and HGC27 cells treated with METTL14 shRNAs. **l**, **m** VAMP3 mRNA expression levels were measured by qRT‒PCR in AGS and HGC27 cells with METTL14 knockdown treated with actinomycin D (2 µg/mL) at the indicated time points. **n**, **o** qRT‒PCR and Western blot analysis of VAMP3 expression levels in AGS and HGC27 cells with IGF2BP2 knockdown. **p** A RIP assay was used to detect the enrichment of IGF2BP2 binding to VAMP3 m^6^A modification sites. **q** Western blotting was used to detect VAMP3 expression in AGS cells transfected with wild-type METTL14 plasmid or IGF2BP2 knockdown. *p < 0.05, **p < 0.01, ***p < 0.001.

To characterize potential downstream targets involved in the m^6^A-regulated occurrence and development of GC mediated by *H. pylori*, we identified 103 genes that overlapped between the m^6^A-downregulated and differentially expressed genes and selected the top 8 genes with the most significant expression differences (Fig. 4a). Then, we validated the mRNA levels of these candidate genes in *H. pylori*-treated AGS cells and METTL14 knockdown GC cell lines (AGS and HGC27). Only VAMP3 was consistently regulated by *H. pylori* and METTL14 in GC cells (Fig. 4b–e and Supplementary Fig. 1b, c). MeRIP-seq revealed that the m^6^A abundance in VAMP3 mRNA was significantly depressed upon *H. pylori* treatment (Supplementary Fig. 1d). The MeRIP-qPCR assay was performed to test whether METTL14 could regulate the m^6^A modification level of VAMP3 mRNA, and the results showed that METTL14 overexpression upregulated the m^6^A modification level of VAMP3 mRNA (Fig. 4f). Moreover, we found that METTL14 overexpression led to the upregulation of VAMP3 expression. Compared to wild-type METTL14, the METTL14-R298P mutant lost the ability to upregulate VAMP3 expression (Fig. 4g, h). Therefore, we selected VAMP3 as a candidate target for METTL14-mediated m^6^A modification in GC cells.

To investigate the molecular mechanism by which METTL14 mediates VAMP3 m^6^A modifications and regulates its expression, we first explored whether METTL14 regulated VAMP3 mRNA expression through its m^6^A motif by a dual-luciferase reporter assay. Based on these results of MeRIP-seq analysis and using SRAMP software to predict the m^6^A site of VAMP3 mRNA, we replaced the N6-methylated adenosine (A) in the m^6^A consensus motif of VAMP3 mRNA with T(thymine) and established a mutant VAMP3 plasmid (Fig. 4i and Supplementary Fig. 1e). The results of the dual-luciferase reporter gene assay showed that METTL14 knockdown significantly reduced the luciferase activity of the wild-type VAMP3 plasmid, while the luciferase activity of the mutant VAMP3 plasmid was not significantly changed (Fig. 4j, k). We then investigated whether the m^6^A modification affected the stability of VAMP3 mRNA by detecting the half-life of VAMP3 mRNA through an actinomycin D assay. The results showed that the half-life of VAMP3 mRNA was significantly shortened in both AGS and HGC27 cells when METTL14 was downregulated (Fig. 4l, m). These results suggest that METTL14 mediates the m^6^A modification of VAMP3 mRNA, enhancing its stability and thus regulating its expression.

It is known that the main mechanism by which m^6^A modification affects mRNA fate is through the recruitment of m^6^A readers. IGF2BPs, including IGF2BP1/2/3, play a pivotal role in augmenting the stability of targeted mRNAs through a m^6^A-dependent mechanism [7]. Our previous results indicated that METTL14 mediated m^6^A modification of VAMP3 mRNA and enhanced its stability, so we speculated IGF2BPs might be involved as readers of m^6^A-modified VAMP3. We first explored which molecule among IGF2BPs promoted VAMP3 stability and found that IGF2BP2 knockdown resulted in a significant reduction in VAMP3 expression in both AGS and HGC27 cells, while IGF2BP1 and IGF2BP3 did not (Fig. 4n, o and Supplementary Fig. 1f, g). RIP experiments showed that IGF2BP2 could bind to VAMP3 mRNA (Fig. 4p). In addition, western blot results showed that METTL14 overexpression significantly upregulated VAMP3 expression, but IGF2BP2 knockdown diminished the up-regulation of VAMP3 caused by METTL14 overexpression (Fig. 4q). Taken together, these results suggest that *H. pylori*-induced downregulation of METTL14 reduces the m^6^A modification levels of VAMP3 and further diminishes IGF2BP2-dependent mRNA stability, thereby inhibiting VAMP3 expression in GC cells.

### *H. pylori* accelerates GC malignant progression by downregulating METTL14/VAMP3

**Fig. 5:**
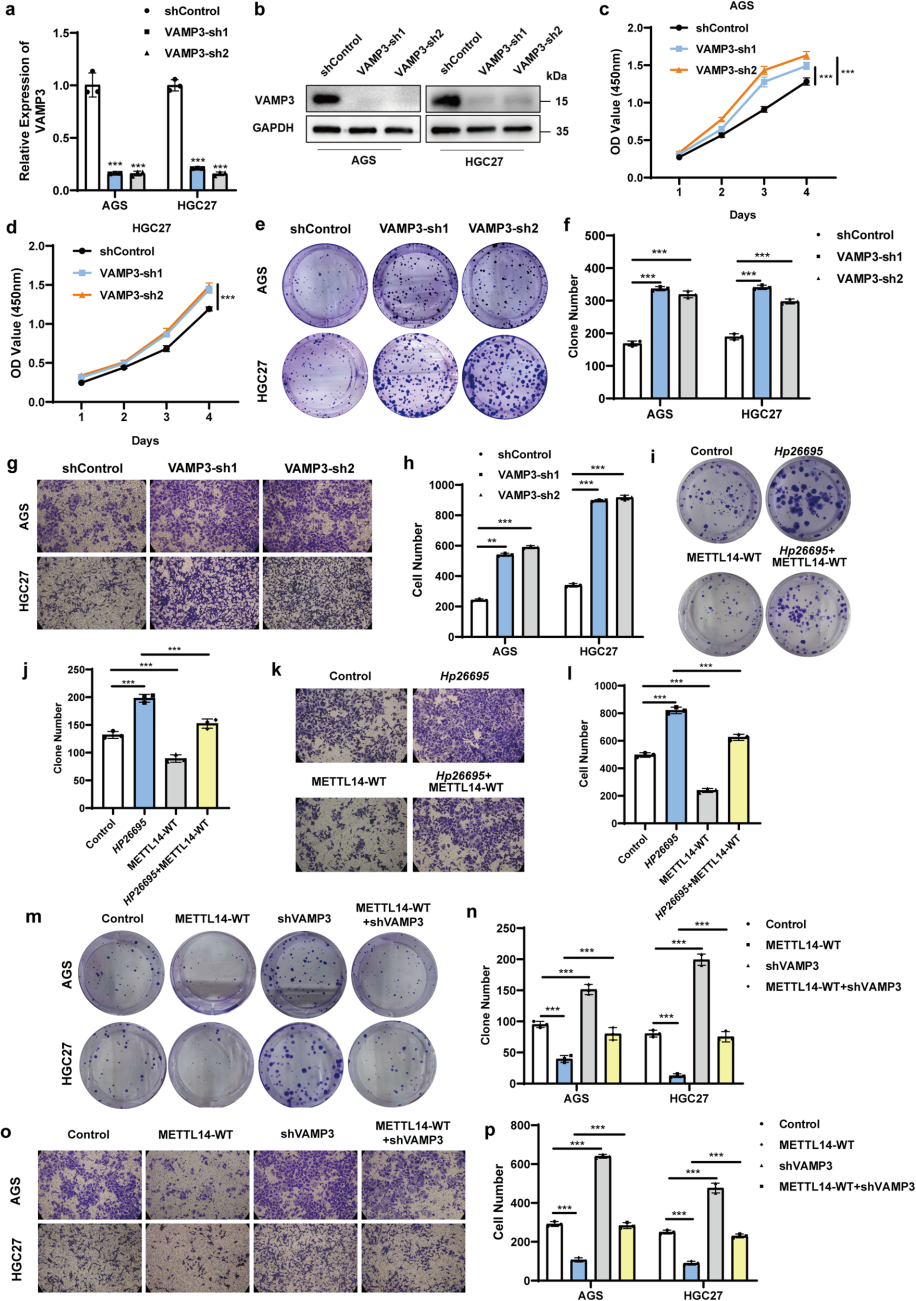
*H. pylori* accelerates GC malignant progression by downregulating METTL14/VAMP3. **a, b** qRT‒PCR and Western blotting were performed to detect VAMP3 expression levels in AGS and HGC27 cells with VAMP3 knockdown. CCK-8 (**c, d**) and colony formation (**e**) assays were used to detect the effect of METTL14 knockdown on GC cell proliferation. Quantification of the colony formation assay (**f**). Transwell assay (**g**) was used to detect the effect of METTL14 knockdown on GC cell migration. Quantification of the Transwell assay (**h**). AGS cells transfected with wild-type METTL14 plasmid or control were treated with *Hp26695* at MOI = 100 for 6 h. Colony formation (**i**) and Transwell (**k**) assays were performed. Quantification of the colony formation (**j**) and Transwell (**l**) assays. AGS and HGC27 cells transfected with VAMP3 shRNAs or control were treated with wild-type METTL14 plasmid. Colony formation (**m**) and Transwell (**o**) assays were performed. Quantification of the colony formation (**n**) and Transwell (**p**) assays. *p < 0.05, **p < 0.01, ***p < 0.001.

To characterize the function of VAMP3 in GC, we used two shRNA-VAMP3 plasmids and confirmed the knockdown efficiency at both the mRNA and protein levels (Fig. 5a, b). Knockdown of VAMP3 significantly promoted the proliferation (Fig. 5c, d), clonogenic (Fig. 5e, f) and migration abilities of GC cells (Fig. 5g, h). To further investigate whether *H. pylori* promotes GC progression by regulating METTL14, rescue experiments were performed. The results showed that *H. pylori* promoted the proliferation and migration of AGS cells, while METTL14 overexpression could diminish the proliferation and migration abilities induced by *H. pylori* (Fig. 5i–l). In addition, we conducted rescue experiments to investigate whether METTL14 inhibits GC progression through VAMP3. We found that METTL14 overexpression significantly inhibited the proliferation and migration of AGS cells, while knockdown of VAMP3 could rescue the proliferation and migration abilities inhibited by METTL14 (Fig. 5m–p). Thus, our data suggest that *H. pylori* accelerates GC malignant progression by downregulating METTL14/VAMP3.

### The VAMP3/LC3C-mediated autophagy pathway regulates c-Met recycling

**Fig. 6:**
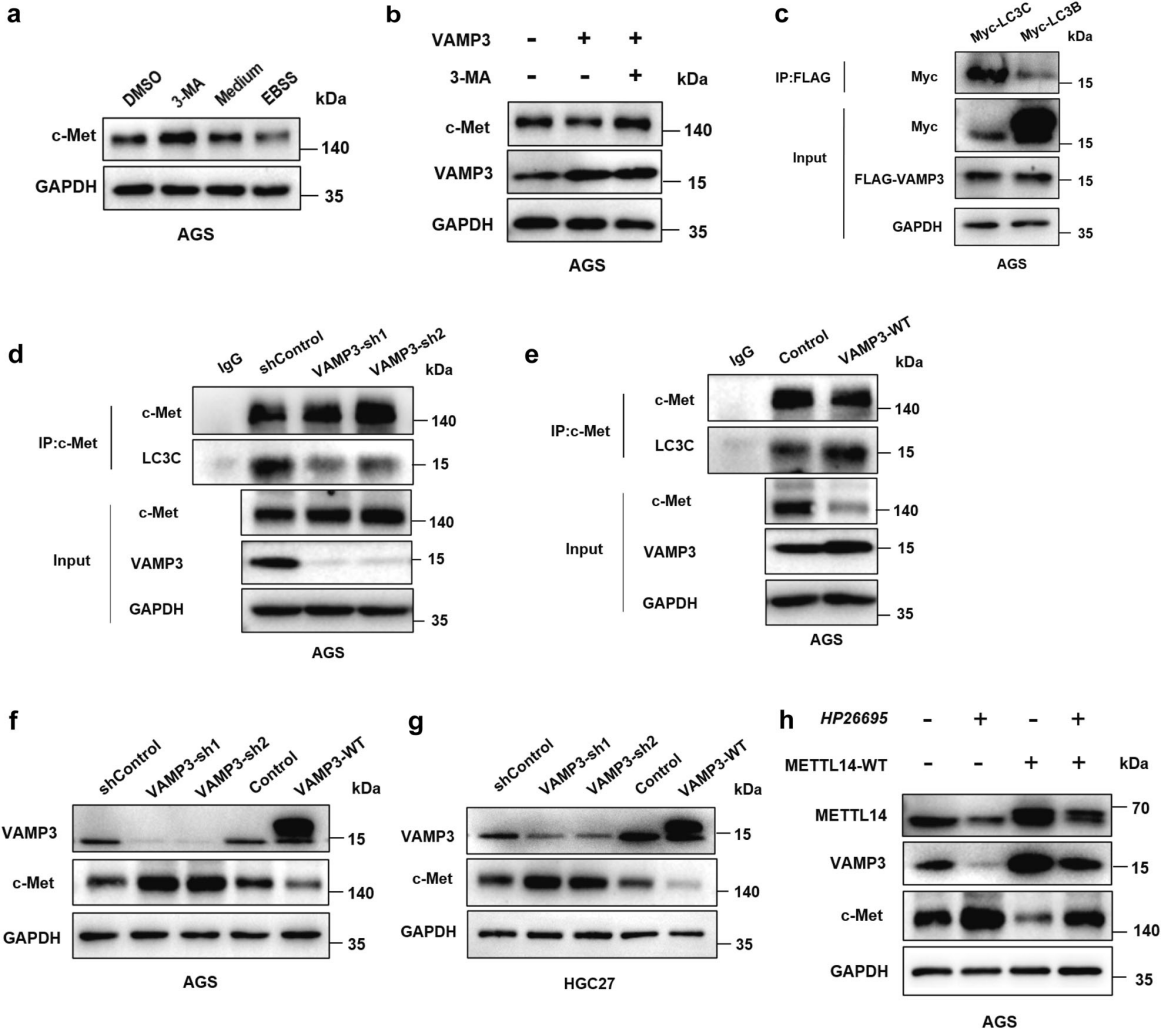
The VAMP3/LC3C-mediated autophagy pathway regulates c-Met recycling. **a** Western blotting was performed to detect c-Met expression in AGS cells treated with 3-MA or EBSS. **b** Western blotting was performed to detect c-Met expression in AGS cells transfected with the VAMP3 plasmid treated with 3-MA or the control for 24 h. **c** Coimmunoprecipitation (Co-IP) of the binding between exogenous VAMP3 and exogenous LC3C or LC3B in AGS cells cotransfected with Flag-VAMP3 plasmid and Myc-LC3C plasmid or Myc-LC3B plasmid. **d** Co-IP of endogenous c-Met and LC3C in AGS cells with VAMP3 knockdown. **e** Co-IP of endogenous c-Met and LC3C in AGS cells overexpressing VAMP3. **f, g** c-Met expression level was measured by Western blot in AGS and HGC27 cells with VAMP3 knockdown or overexpression. **h** Western blotting was performed to detect METTL14, VAMP3 and c-Met expression levels in AGS cells treated with *Hp26695* at MOI = 100 for 12 h or wild-type METTL14 plasmid. *p < 0.05, **p < 0.01, ***p < 0.001.

Recent studies have shown that VAMP3 delivers Met receptor tyrosine kinases (Met-RTKs) to an endocytic compartment where interaction with LC3C occurs, followed by engagement of a c-Met-LC3C complex with ATG9 targeting the initiation site of autophagosomes for the assembly and maturation of autophagic vesicles [18]. However, it is unclear whether c-Met targets autophagic degradation dependent on the LC3C-mediated autophagy pathway in GC cells. We first investigated whether c-Met was degraded through the autophagy pathway and whether VAMP3 was involved in the LC3C-mediated autophagy pathway. The expression of c-Met was upregulated in AGS cells treated with 3-methyladenine (3-MA) but downregulated in AGS cells treated with EBSS (Fig. 6a). Furthermore, treatment with 3-MA reversed the c-Met protein decline caused by VAMP3 overexpression (Fig. 6b). Coimmunoprecipitation showed that VAMP3 selectively interacted with LC3C over LC3B (Fig. 6c). Then, to determine whether c-Met and LC3C interactions were dependent on VAMP3, we knocked down or overexpressed VAMP3 to detect the binding of c-Met to LC3C. The results revealed that VAMP3 is needed for the interaction between c-Met and LC3C (Fig. 6d, e). We found that VAMP3 knockdown significantly upregulated c-Met and VAMP3 overexpression significantly downregulated c-Met (Fig. 6f, g). In addition, c-Met were significantly upregulated in *H. pylori*-treated cells, but METTL14 overexpression diminished the up-regulation of c-Met caused by *H. pylori* (Fig. 6h). Collectively, these results suggest that *H. pylori* accelerates the malignant progression of GC by regulating VAMP3/LC3C-mediated c-Met recycling.

### The METTL14/VAMP3 axis is correlated with *H. pylori*-mediated malignant transformation of the gastric mucosa

**Fig. 7:**
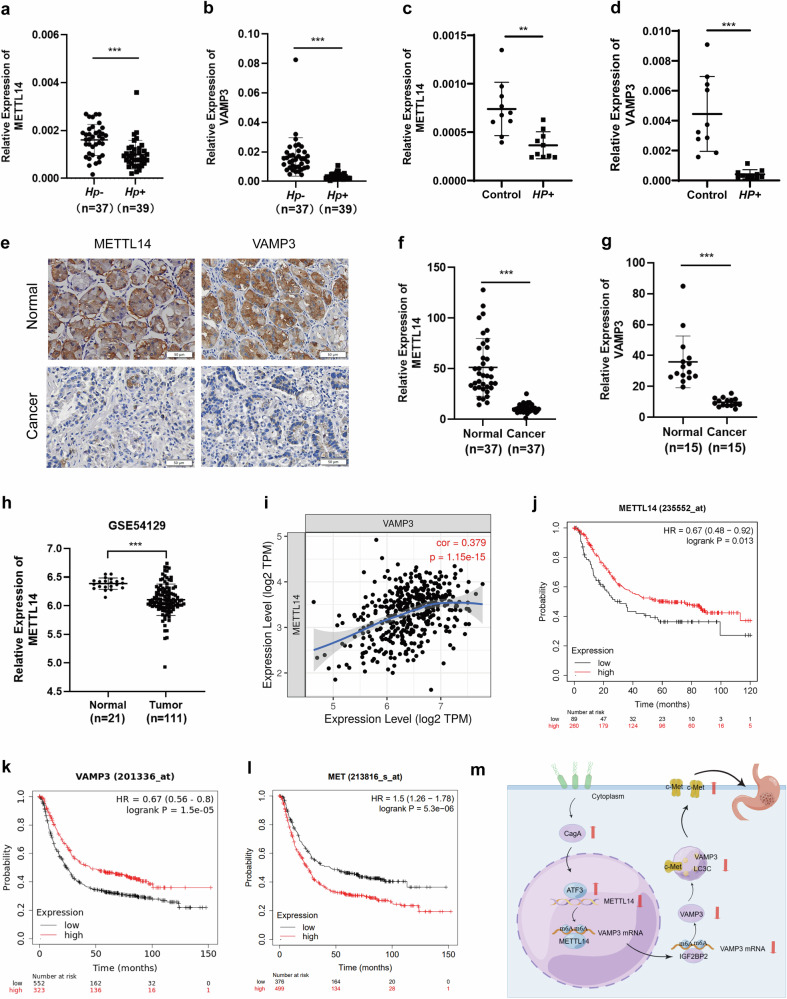
The METTL14/VAMP3 axis is correlated with *H. pylori*-mediated malignant transformation of the gastric mucosa. **a, b** METTL14 and VAMP3 mRNA expression levels were measured by qRT‒PCR in *Hp−* human gastritis tissues (*n* = 37) and *Hp+* human gastritis tissues (*n* = 39). **c, d** qRT‒PCR was used to detect the expression levels of METTL14 and VAMP3 in the *H. pylori*-infected mouse model and control groups (*n* = 10). **e-g** IHC staining for METTL14 and VAMP3 in cancerous tissues of patients with GC and adjacent normal tissues. IHC scores (**f, g**). **h** RNA sequencing database analysis of METTL14 expression in human GC and paired adjacent normal tissues. The data were obtained from GSE54129. **i** Correlation analysis of METTL14 and VAMP3 in GC tissues. **j-l** Kaplan‒Meier analysis of METTL14, VAMP3 and c-Met in the survival of patients with GC in the GEO database. **m** Schematic diagram of the *H. pylori*-induced downregulation of METTL14-mediated VAMP3 m^6^A modifications contributing to malignant transformation of the gastric mucosa (by Figdraw). *p < 0.05, **p < 0.01, ***p < 0.001.

To clarify the correlation between the METTL14/VAMP3 axis and *H. pylori*-mediated malignant transformation of the gastric mucosa, we first identified the expression of METTL14 and VAMP3 in human *Hp−* chronic gastritis tissues and *Hp+* chronic gastritis tissues. The expression levels of METTL14 and VAMP3 in *Hp+* chronic gastritis tissues were much lower than those in *Hp−* chronic gastritis tissues (Fig. 7a, b). Then, we established a gastritis mouse model of *H. pylori* infection to detect the expression of METTL14 and VAMP3. Compared with the control group, METTL14 and VAMP3 were significantly downregulated in the *H. pylori* infection group (Fig. 7c, d). Next, we detected the expression of METTL14 and VAMP3 in cancerous tissues of patients with GC and adjacent normal tissues. METTL14 and VAMP3 expression levels were downregulated notably in cancerous tissues of patients with GC (Fig. 7e–g).

In addition, GEO data analysis revealed that the mRNA levels of METTL14 in cancerous tissues of patients with GC were significantly lower than those in adjacent normal tissues (Fig. 7h). Analysis of the correlation between METTL14 and VAMP3 using TIMER revealed a positive correlation between METTL14 and VAMP3 expression in GC patients [19] (Fig. 7i). Kaplan‒Meier analysis revealed that patients with low levels of METTL14 and VAMP3 had a lower survival rate, and those with high c-Met levels had a lower survival rate [20] (Fig. 7j–l). Taken together, these results suggest that the METTL14/VAMP3 axis is correlated with *H. pylori*-mediated malignant transformation of the gastric mucosa.

## Discussion

*H. pylori*, a spiral-shaped, gram-negative bacterium, plays an important role in the transformation of the gastric mucosa from chronic inflammation to malignancy. Some studies have demonstrated that *H. pylori* promotes the progression of GC by regulating m^6^A modulators and target mRNA m^6^A modification. It is reported that *H. pylori* upregulates FTO-mediated m^6^A modification of genes (such as GLI1 and CD44) to promote the progression of GC [21, 22]. *H. pylori* increases the expression level of METTL3 in GC cells, thereby leading to METTL3-mediated m^6^A modification of STAT5A to promote the progression of GC [23]. In this study, we found that infection with *H. pylori* significantly downregulated METTL14 to promote the progression of GC.

ATF3 is a stress-induced transcription factor. ATF3 can act as a transcriptional repressor by forming a homodimer. ATF3 can also cooperatively form heterodimers with other ATF/CERB family proteins or CCAAT/enhancer-binding protein (C/EBP) family proteins producing inhibitory or stimulatory effects [24, 25]. ATF3 can be upregulated in response to a variety of cellular stress situations, including DNA damage, cell damage, and oncogenic stimuli [26–28]. The functions of ATF3 in glucose metabolism, adipocyte differentiation, regulation of immune responses, host defences and cancers have been extensively studied [29]. It is reported that *H. pylori* upregulates the expression of ATF3 in GC cells [30, 31]. However, the mechanism by which ATF3 regulates m^6^A-related proteins on the occurrence and development of GC mediated by *H. pylori* has not been reported. In this study, we found that *H. pylori* repressed METTL14 by upregulating the transcriptional factor ATF3. However, whether ATF3 exerts its inhibitory effect on METTL14 transcription through the formation of homodimers or heterodimers still requires further exploration.

In the m^6^A methyltransferase complex, METTL3 functions as a catalytic subunit, and METTL14 functions as an RNA-binding scaffold that recognizes the substrate and promotes the stabilization of METTL3 [6]. The evidence accumulated in recent years indicates that METTL14 inhibits the malignant progression of various cancer cells, such as colorectal cancer [32, 33], liver cancer [34, 35] and renal cell carcinoma [36, 37]. In GC, METTL14-mediated m^6^A modification of circORC5 suppresses GC progression by regulating miR-30c-2-3p/AKT1S1 axis [38]. CircUGGT2 downregulation by METTL14-mediated m^6^A modification suppresses GC progression and cisplatin resistance through interaction with miR-186–3p/MAP3K9 axis [39]. In addition, our previous research shows that Lnc-PLCB1 is stabilized by METTL14 induced m^6^A modification and inhibits *H. pylori* mediated GC by destabilizing DDX21 [16]. However, the m^6^A modification of important downstream target mRNA regulated by METTL14 in GC remains unclear. In this study, we found that *H. pylori* promoted GC progression by reducing METTL14-mediated VAMP3 m^6^A modification.

m^6^A readers can specifically recognize and bind to m^6^A modification sites. They are generally divided into three major classes: IGF2BPs, YT521 - B homology (YTH) domain family proteins, and a group of heterogeneous ribonucleoproteins (hnRNP). IGF2BPs increase the stability of m^6^A modification transcripts by recruiting RNA stabilizers (such as ELAVL1, MATR3 and PABPC1), and probably promote the translation of target mRNAs by recruiting eukaryotic translation initiation factor (eIF) proteins [7, 40]. In addition, studies have shown that IGF2BPs stabilize m^6^A modification targets through a miRNA-dependent mechanism [41–43]. Some studies indicate that IGF2BP2 increases the stability of m^6^A-containing mRNA, such as CSF2 [44], HMGA1 [45], ZEB1 [46]and STAT5A [23], promoting GC progression. Consistent with these studies, we found that IGF2BP2 bound to VAMP3 through a m^6^A-dependent manner, enhancing VAMP3 mRNA stability and expression. However, the detailed mechanism by which IGF2BP2 enhances VAMP3 stability requires further exploration.

LC3B is the most widely used marker for autophagosomes [47], while LC3C is less studied and is associated with selective autophagic pathways [48]. The LC3C-endocytic-associated-pathway (LEAP) is a subset of LC3C that localizes to peripheral endosomes in a process regulated by the serine–threonine kinase TBK1. Endosomal LC3C engages with PM-derived cargo, including Met-RTK and transferrin receptor 1 (TfR), and recruits these proteins for autophagic degradation. VAMP3 is needed for the interaction of LC3C with PM-derived cargo [49]. Our study shows that VAMP3 downregulation leads to the uncoupling of c-Met from autophagic vesicles, the subsequent recycling of c-Met to the cell membrane, and ultimately the upregulation of c-Met in GC cells.

c-Met is a unique subfamily of receptor tyrosine kinases (RTK), which is only activated by hepatocyte growth factor (HGF) and plays essential roles in controlling many crucial cellular processes, such as cell proliferation, survival, motility and morphogenesis [50]. c-Met, as an oncogene, plays an important role in promoting the occurrence and development of GC [51–53]. In recent years, it has served as an important biomarker candidate and therapeutic target for GC. It is reported that c-Met is significantly increased in the presence of *H. pylori* infection in dysplasia and GC samples [54]. However, there are very few reports on the mechanism by which *H. pylori* upregulates c-Met expression. Our research provides evidence that *H. pylori* upregulates the expression of c-Met through the VAMP3/LC3C-mediated autophagy pathway.

ERK belongs to the family of mitogen-activated protein kinases (MAPK). This family functions in signal cascades and transmits extracellular signals to intracellular targets. Therefore, the MAPK cascade is a central signal transduction pathway for regulating fundamental processes, including cell proliferation, differentiation, and stress responses [55–57]. It has been reported that *H. pylori* activated c-Met/ERK signalling to promote the malignant transformation of GC [58, 59]. We also found that VAMP3 knockdown significantly upregulated c-Met and p-ERK in GC cells. But the mechanism by which c-Met activates the ERK signalling pathway still requires further investigation.

In conclusion, *H. pylori* reduces METTL14-mediated VAMP3 m^6^A modification and promotes the development of GC through the VAMP3/LC3C-mediated autophagy pathway to regulate c-Met recycling (Fig. 7m). Our work reveals a novel mechanism of *H. pylori* mediated gastric carcinogenesis which helps to expand our understanding of epigenetic regulation in the progression of GC.

## Materials and methods

### Cell culture and *H. pylori* culture

The GC cell lines (AGS and HGC27) were purchased from the Cell Resource Center at the Shanghai Institute of Biochemistry and Cell Biology at the Chinese Academy of Sciences (Shanghai, China). HGC27 cells were cultured in RPMI-1640 medium (M&C GENE, China) plus 10% (v/v) foetal bovine serum (FBS). AGS cells were cultured in F12K medium plus 12% (v/v) FBS. The medium and FBS were purchased from Gibco/Life Technologies (Grand Island, NY, USA). The cell lines were cultured at 37 °C in a humidified 5% CO_2_ atmosphere. *H. pylori* strains 11637 (*Hp11637*) and 26695 (*Hp26695*) were cultivated on Brucella agar plates containing 5% defibrinated sheep blood at 37 °C under microaerobic conditions (5% O_2_, 10% CO_2_ and 85% N_2_).

### Transfection

The sequences of small interfering RNAs (siRNAs) are listed in Supplementary Table 1. The shMETTL14, METTL14-WT, METTL14-MUT, shVAMP3, VAMP3, Myc-LC3C and Myc-LC3B plasmids were purchased from Miaolingbio (China). The siRNAs and plasmids were transiently transfected with jetRPIME (Polyplus, France). METTL14-WT lentivirus with PuroR was purchased from Hanbio (China).

### RNA extraction and qRT‒PCR

TRIzol (Vazyme, China) was used to extract total RNA from GC cells and tissues. The RNA was reverse transcribed into cDNA with an Evo M-MLV RT kit (AG, China). qRT–PCR was conducted using a SYBR Green Pro Taq HS qPCR kit (AG, China) and a CFX96 Real-time PCR System (Bio-Rad, USA). The primer sequences are listed in Supplementary Table 1.

### Protein extraction and Western blot

RIPA buffer (high) (Solarbio, China) containing protease inhibitor was used to extract total proteins from GC cells. Total proteins were separated by SDS‒PAGE, transferred to PVDF membranes, incubated with 5% skim milk for 1 h, specific primary antibodies overnight at 4 °C, and corresponding secondary antibodies for 1 h, and finally detected by an ECL kit (Millipore, USA). The antibodies are listed in Supplementary Table 1.

### Dual-luciferase reporter assays

For the METTL14 promoter activity assay, the 1000 bp upstream of the METTL14 promoter was inserted into the promoter region of the pGL3 vector. For m^6^A reporter assays, the mRNA fragments of VAMP3 containing the WT m^6^A motifs, as well as the mutated motifs, were inserted behind the F-Luc of the pMIR-GLO vector.

### Proliferation and migration assays

GC cells were transfected with plasmids or siRNA for 48 h before the following experiments were performed. For the CCK-8 assay, AGS and HGC27 cells were seeded in 96-well plates at 3×10^3^ cells/well and 2.5×10^3^ cells/well, respectively. After incubation for 24, 48 and 72 h, CCK8 reagent (APExBIO, USA) was added, and the absorbance value at 450 nm was detected. For the EdU assay, AGS and HGC27 cells were seeded in 96-well plates at 1.5×10^5^ cells/well and 1×10^5^ cells/well, respectively. After incubation for 12 h, EdU assays were performed using the BeyoClick^TM^ EdU Cell Proliferation Kit with Alexa Fluor 488 (Beyotime, China). For the colony formation assay, AGS and HGC27 cells were seeded in 6-well plates at 900 cells/well. After incubation of AGS cells for 14 days and HGC27 cells for 10 days, cells were fixed with methanol for 20 min and stained with 0.05% crystal violet staining solution for 20 min. For the Transwell assay, 600 μl medium containing 20% FBS was added to 24-well plates. Transwell chambers were seeded with 5×10^4^ AGS cells and 4×10^4^ HGC27 cells with 100 μl FBS-free medium, and Transwell chambers were immersed in medium. After incubation of AGS and HGC27 cells for 48 h, cells were fixed with methanol for 20 min and stained with 0.05% crystal violet staining solution for 20 min.

### Animal experiments

Animal experiments were approved and guided by the Ethics Committee of Shandong University School of Medicine (Jinan, China). Four-week-old female BALB/c nude mice (Beijing Vital River Laboratory Animal Technology Co., Ltd., China) were randomly divided into two groups with 6 mice in each group. METTL14-WT HGC27 cells (5×10^6^ cells/mouse) and negative control HGC27 cells were injected into the subcutaneous axilla of nude mice. The length and width of each tumour were measured with a Vernier calliper. The tumour volume was calculated as (length×width^2^)/2. Finally, each tumour was removed and weighed. METTL14-WT HGC27 cells (2.5×10^6^ cells/mouse) and negative control HGC27 cells were intravenously injected into nude mice. After 25 days, mouse livers were removed and weighed, followed by HE staining experiments.

### *H. pylori*-infected mouse model

Four-week-old female BALB/c nude mice (Beijing Vital River Laboratory Animal Technology Co., Ltd., China) were randomly divided into two groups. One group of mice was treated with antibiotics for gut sterilization for 7 days. Then, the mice were inoculated with the *H. pylori* strain suspension (10^8^/inoculum) every other day for a total of three times. After 2 months, the mice were sacrificed, and stomach tissues were collected for further study.

### m^6^A sequencing (MeRIP-seq) assays

m^6^A sequencing technology services were provided by Cloudseq Biotech, Inc. (Shanghai, China). The methods were as follows: immunoprecipitation of m^6^A RNA was performed using a GenSeq^TM^ m^6^A-MerIP kit (GenSeq). The NEBNext® Ultra II Directional RNA Library Prep kit was used for RNA sequencing library construction on input RNA samples without immunoprecipitation and IP RNA samples after immunoprecipitation. Library quality was checked by a BioAnalyzer 2100 (Agilent). High-throughput sequencing was performed in 150 bp paired-end mode on an Illumina HiSeq sequencer.

### MeRIP-qPCR

MeRIP assays were performed using the MeRIP kit (BersinBio, China). The methods were as follows: RNA from 1×10^8^ AGS cells was extracted, and the RNA was incubated with m^6^A antibody on beads overnight at 4 °C. After digestion, RNA was extracted and analysed by qRT‒PCR. The primer sequences are listed in Supplementary Table 1.

### RIP assays

A RIP kit (Millipore, MA) was used to perform the RIP assays. The methods were as follows: total RNA was extracted, and the RNA was incubated with IGF2BP2 antibody on beads overnight at 4 °C. After digestion, RNA was extracted and analysed by qRT‒PCR.

### Actinomycin D assays

AGS and HGC27 cells were cultured overnight. At different times (0, 1.5 and 3 h), the transcription inhibitor actinomycin D (2 μg/mL) was added, and the cells were harvested for RNA extraction.

### Immunoprecipitation assays

Total protein was extracted with RIPA lysis buffer (weak) (Beyotime, China) containing protease inhibitors. Proteins were incubated with antibodies attached to Protein A/G Magnetic Beads (MCE, USA) at 4 °C overnight. After washing the magnetic beads with PBST, SDS‒PAGE loading buffer (Beyotime, China) was added, and the beads in the buffer were heated for 10 min at 100 °C.

### Immunohistochemistry (IHC)

After deparaffinization, rehydration and antigen retrieval, paraffin-embedded sections of tissue were treated with H_2_O_2_ for 20 min, blocked with goat serum for 30 min, and incubated with the primary antibody overnight at 4 °C. The sections were incubated with corresponding secondary antibodies for 1 h and detected using a DAB staining kit (ZSGB-Bio; OriGene Technologies, Inc., China).

### Patients and specimens

A total of 37 *Hp-* human gastritis tissues, 39 *Hp+* human gastritis tissues, and 37 surgically resected gastric cancer tissues and adjacent tissues were collected from Qilu Hospital of Shandong University. The informed consent of patients has been acquired. This study was approved by the Ethics Committee of Shandong University School of Medicine (Jinan, China) and was conducted in accordance with the Declaration of Helsinki.

### Data deposition

The MeRIP-seq data used in this study have been uploaded to the Gene Expression Omnibus (GEO) with the accession code GSE231672.

### Statistical analysis

Statistical analysis was conducted using GraphPad Prism 8.0 and SPSS. All data are presented as the mean ± SD of at least three biological replicates. ANOVA with an appropriate post hoc correction was used for comparisons among multiple groups. Student’s *t* test was generally used to analyse the differences between two groups, but when the variances differed, the Mann–Whitney *U* test was used. *P* < 0.05 (two-tailed) was considered to indicate a statistically significant difference.

## Supplementary information

Original Data

Supplementary Material

Supplementary Material

## Data Availability

All the presenting data are available within the article or supplementary files.
