# Supplementary figures and images for: Helicobacter pylori reduces METTL14-mediated VAMP3 m6A modification and promotes the development of gastric cancer by regulating LC3C-mediated c-Met recycling
Source: Cell Death Discov. 2025 Jan 18;11:13. doi: 10.1038/s41420-025-02289-z (PMC11742886; doi:10.1038/s41420-025-02289-z)

**Figure1**

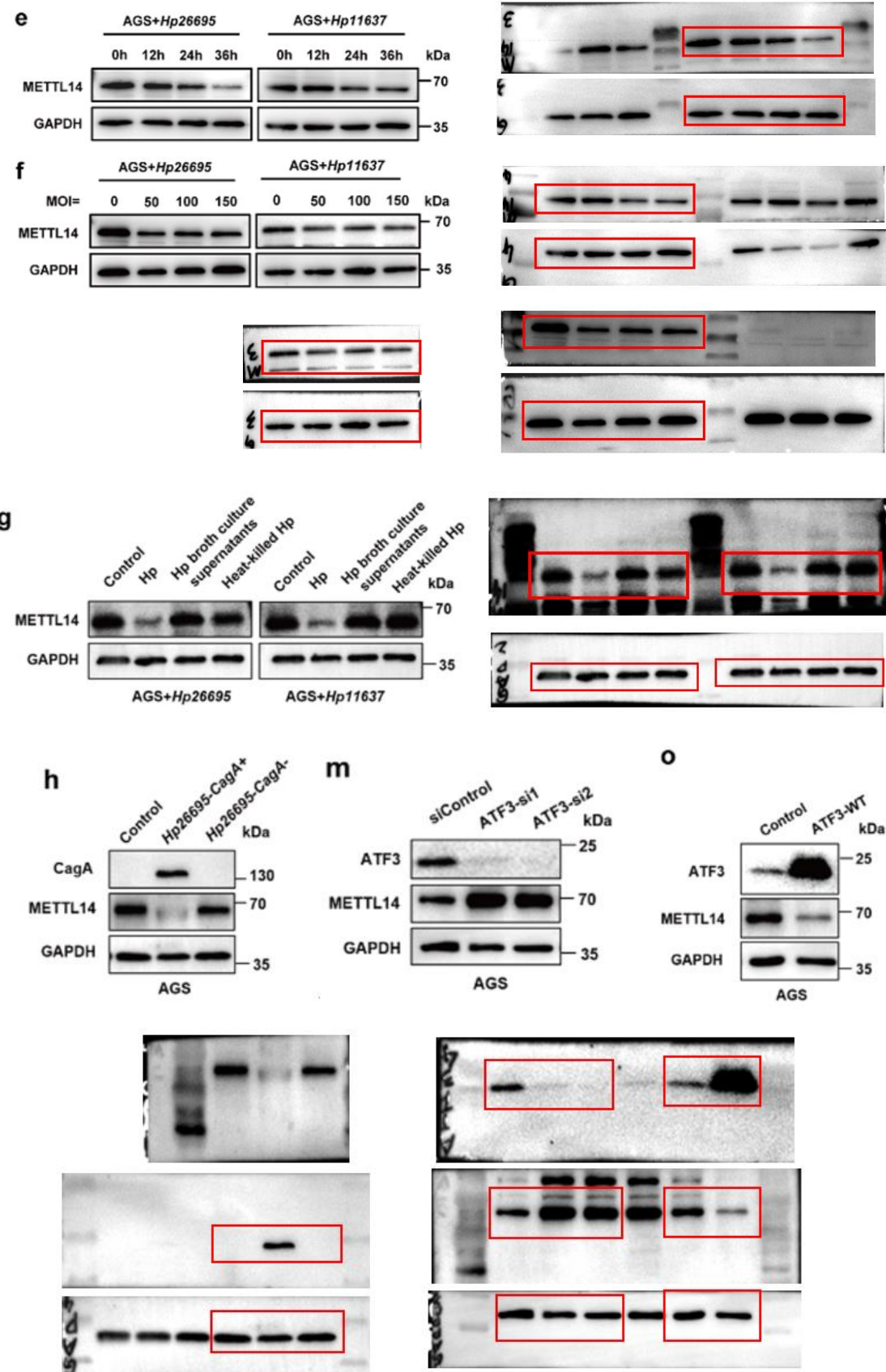

k

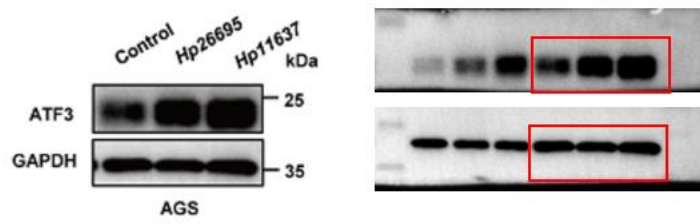

Figure2

c

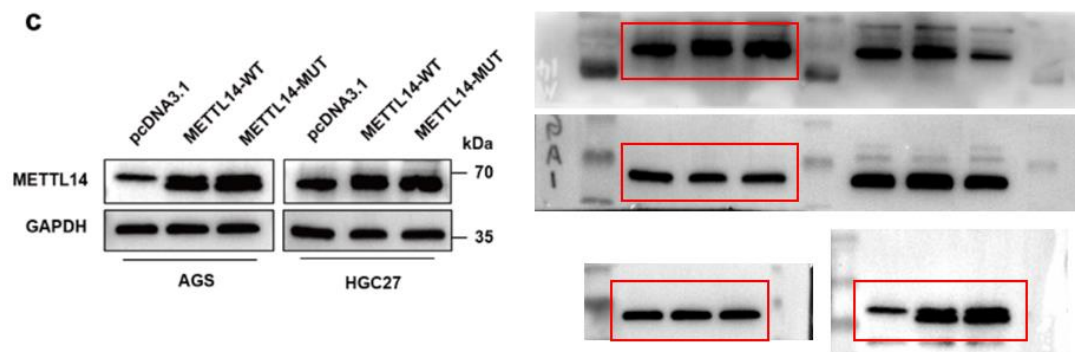

m

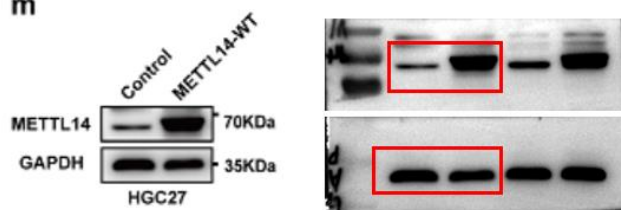

Figure4

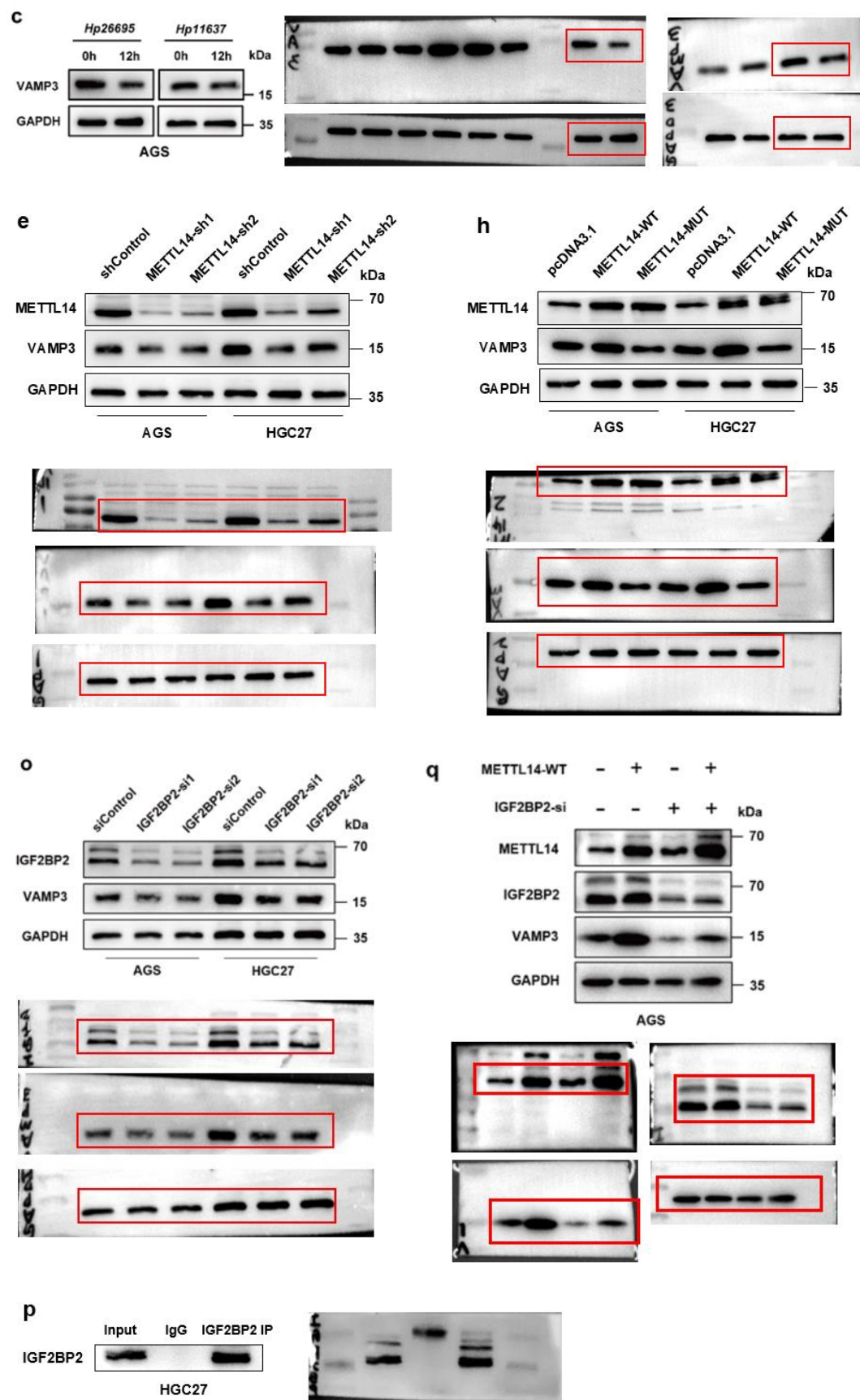

Figure5

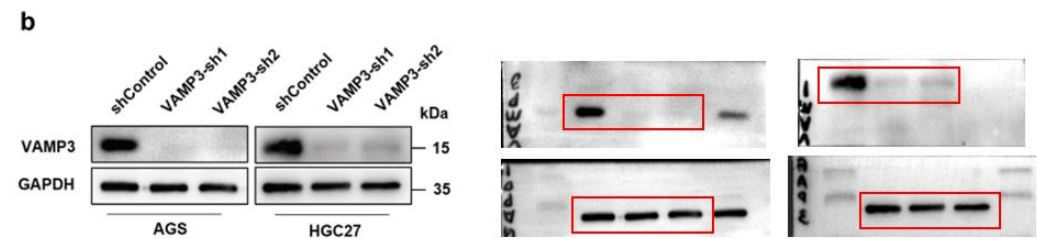

Figure6

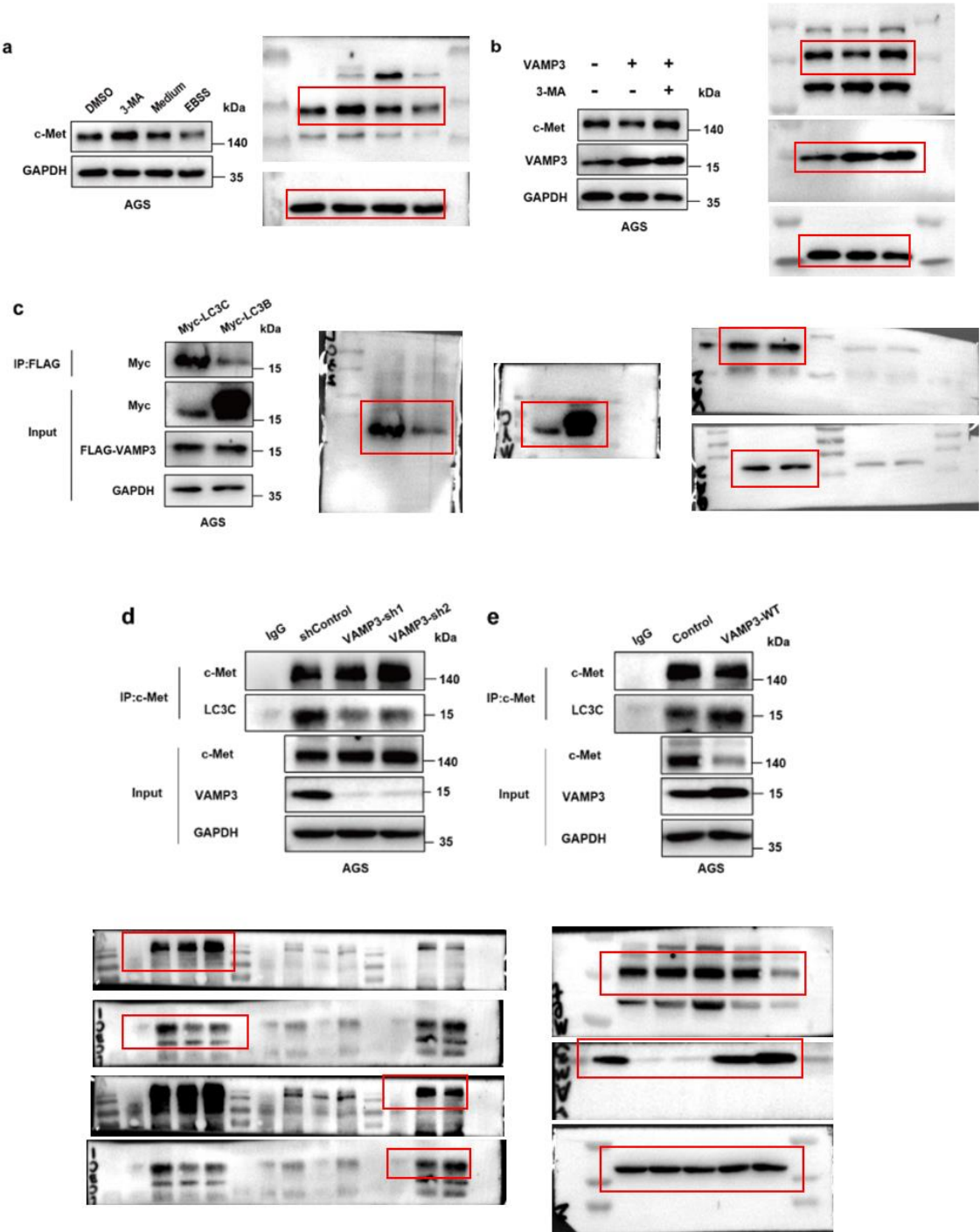

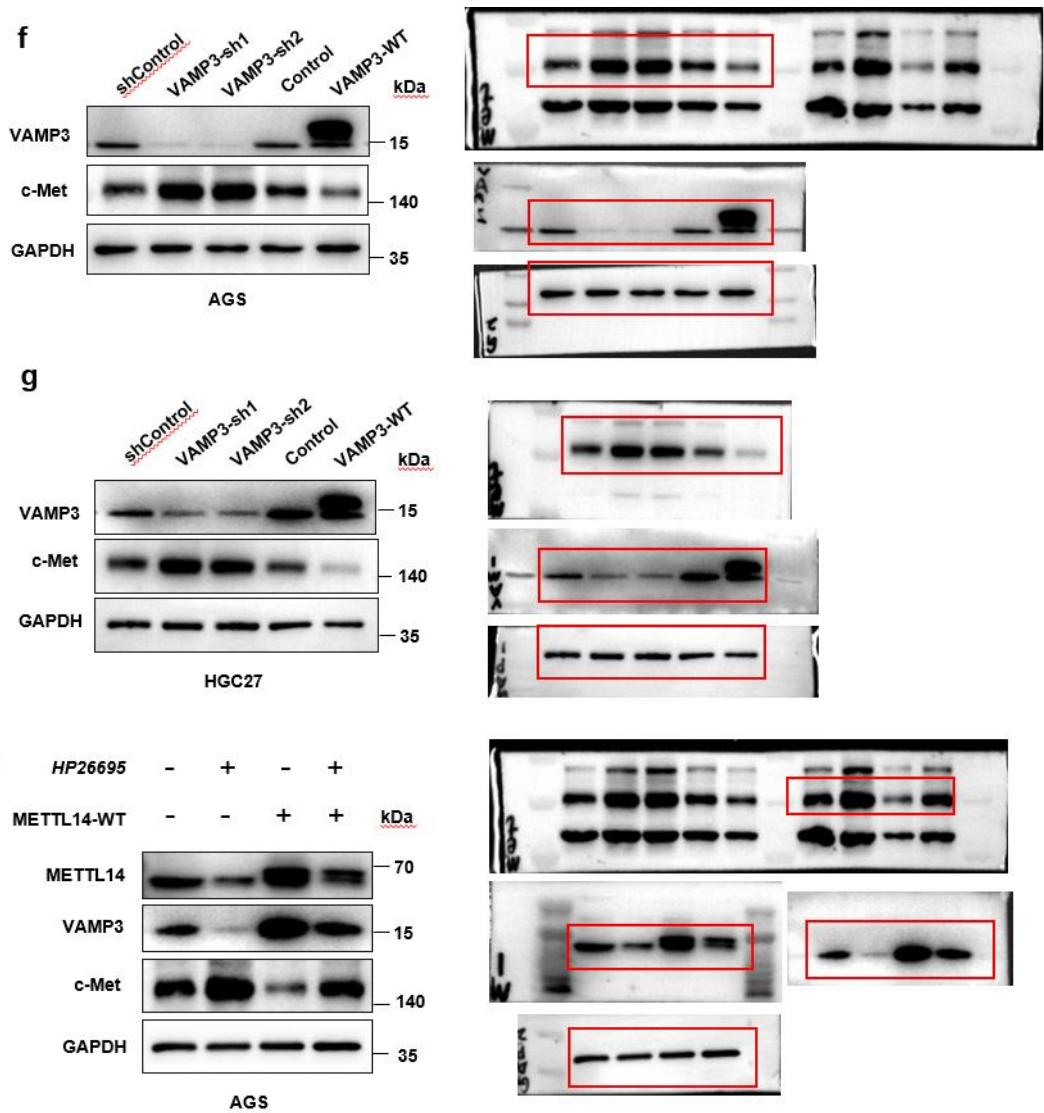

Supplementary Figure 2

b

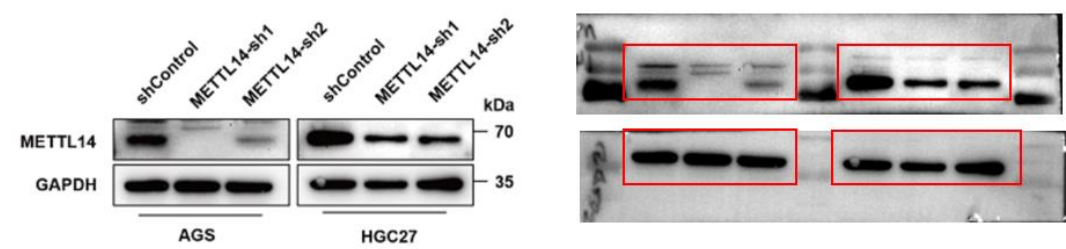

Supplement: Supplementary file 1 — Original Data [file 41420_2025_2289_MOESM1_ESM.pdf]
