# Supplementary material for: Helicobacter pylori reduces METTL14-mediated VAMP3 m6A modification and promotes the development of gastric cancer by regulating LC3C-mediated c-Met recycling
Source: Cell Death Discov. 2025 Jan 18;11:13. doi: 10.1038/s41420-025-02289-z (PMC11742886; doi:10.1038/s41420-025-02289-z)
Supplement: Supplementary file 2 — Supplementary Material [file 41420_2025_2289_MOESM2_ESM.docx]

**
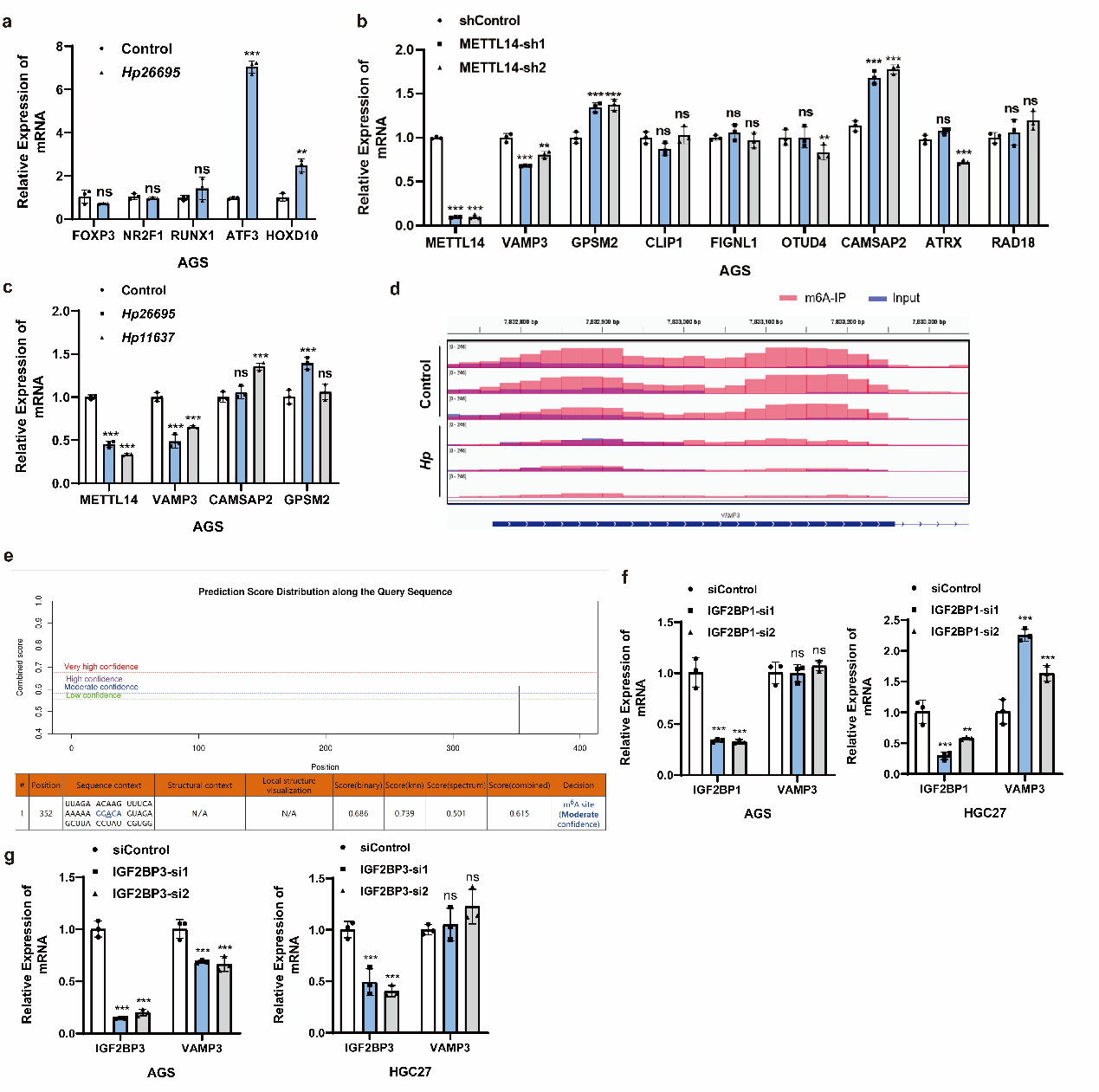
Supplementary Figure 1**

**a** qRT-PCR analysis in AGS cells treated with *Hp26695* at MOI = 100 for 12 h. **b** mRNA was measured by qRT-PCR in AGS cells with METTL14 knock-down. **c** mRNA was measured by qRT-PCR in AGS cells treated with *Hp26695* and *Hp11637* at MOI = 100 for 12 h. **d** Distribution of m^6^A peaks on VAMP3 mRNA transcripts in AGS cells treated with *Hp26695* and the control. **e** The SRAMP website was used to predict the m^6^A modification site of VAMP3. **f** qRT-PCR was used to detect VAMP3 expression levels in AGS and HGC27 cells with IGF2BP1 knockdown. **g** qRT-PCR was used to detect VAMP3 expression levels in AGS and HGC27 cells with IGF2BP3 knockdown. *p < 0.05, **p < 0.01, ***p < 0.001.

**
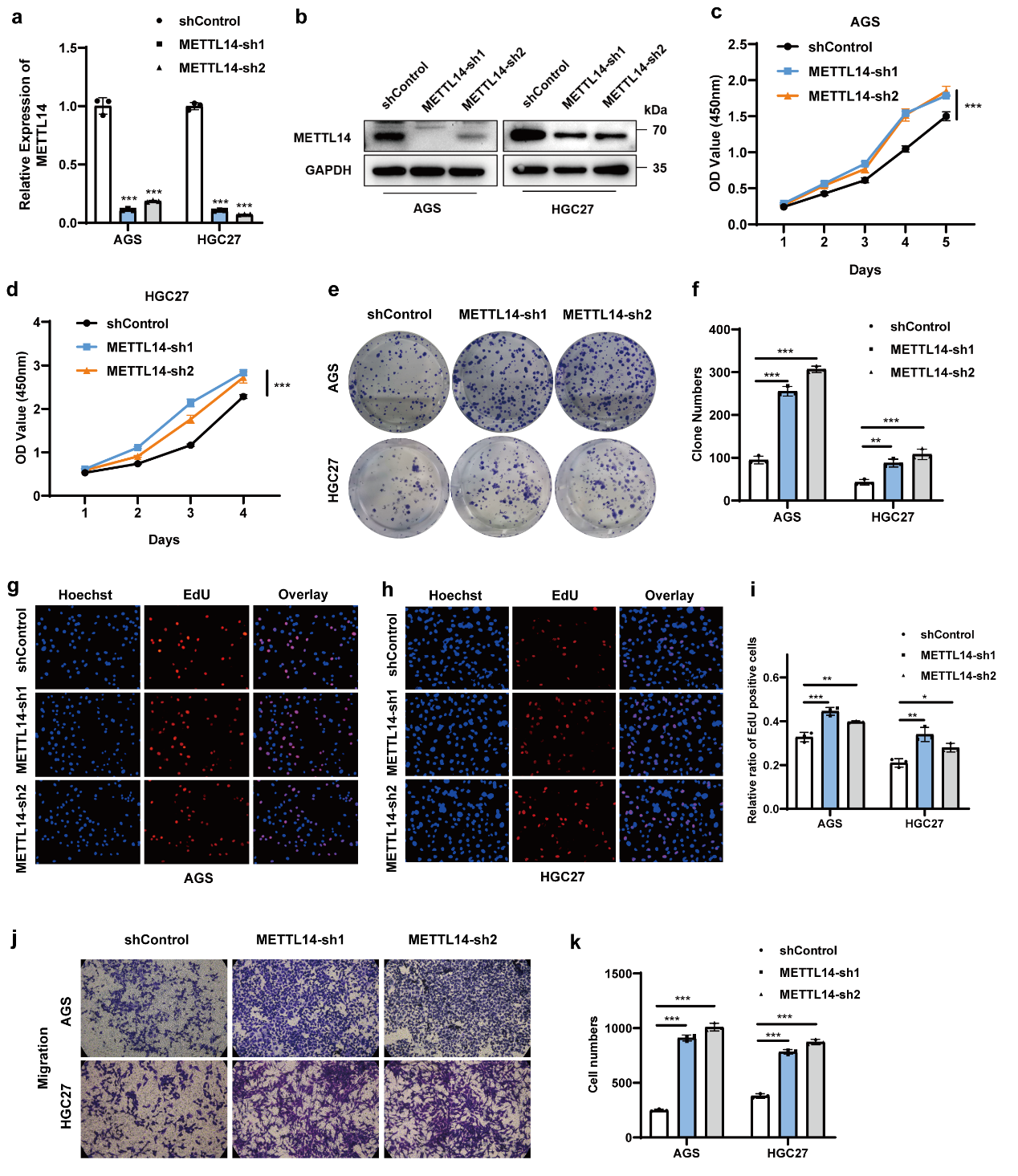
Supplementary Figure 2**

**a, b** qRT-PCR and Western blotting were used to detect METTL14 expression levels in AGS and HGC27 cells with METTL14 knockdown. **c-i** CCK-8 (**c, d**), colony formation (**e**) and EdU (**g, h**) assays were used to detect the effect of METTL14 knockdown on GC cell proliferation. Quantification of the colony formation (**f**) and EdU (**i**) assays. **j, k** Transwell assay (**j**) was used to detect the effect of METTL14 knockdown on GC cell migration. Quantification of the Transwell assay (**k**). *p < 0.05, **p < 0.01, ***p < 0.001.
